# Melatonin Induces Autophagy via Reactive Oxygen Species-Mediated Endoplasmic Reticulum Stress Pathway in Colorectal Cancer Cells

**DOI:** 10.3390/molecules26165038

**Published:** 2021-08-20

**Authors:** Kian Chung Chok, Rhun Yian Koh, Ming Guan Ng, Pei Ying Ng, Soi Moi Chye

**Affiliations:** 1 School of Health Science, International Medical University, Kuala Lumpur 57000, Malaysia; chok.kianchung@student.imu.edu.my (K.C.C.); NG.MINGGUAN@student.imu.edu.my (M.G.N.); 2 Division of Biomedical Science and Biotechnology, School of Health Science, International Medical University, Kuala Lumpur 57000, Malaysia; rhunyian_koh@imu.edu.my; 3 School of Postgraduate, International Medical University, Kuala Lumpur 57000, Malaysia; NG.PEIYING@student.imu.edu.my

**Keywords:** melatonin, autophagy, colorectal cancer cells, reactive oxygen species, endoplasmic reticulum stress

## Abstract

Even though an increasing number of anticancer treatments have been discovered, the mortality rates of colorectal cancer (CRC) have still been high in the past few years. It has been discovered that melatonin has pro-apoptotic properties and counteracts inflammation, proliferation, angiogenesis, cell invasion, and cell migration. In previous studies, melatonin has been shown to have an anticancer effect in multiple tumors, including CRC, but the underlying mechanisms of melatonin action on CRC have not been fully explored. Thus, in this study, we investigated the role of autophagy pathways in CRC cells treated with melatonin. In vitro CRC cell models, HT-29, SW48, and Caco-2, were treated with melatonin. CRC cell death, oxidative stress, and autophagic vacuoles formation were induced by melatonin in a dose-dependent manner. Several autophagy pathways were examined, including the endoplasmic reticulum (ER) stress, 5′–adenosine monophosphate-activated protein kinase (AMPK), phosphoinositide 3-kinase (PI3K), serine/threonine-specific protein kinase (Akt), and mammalian target of rapamycin (mTOR) signaling pathways. Our results showed that melatonin significantly induced autophagy via the ER stress pathway in CRC cells. In conclusion, melatonin demonstrated a potential as an anticancer drug for CRC.

## 1. Introduction

Colorectal cancer (CRC) ranks third in incidence and second in cause of cancer death worldwide, with more than 1.9 million new cases and 935,000 deaths in the year 2020 [1]. The number of deaths caused by CRC worldwide is predicted to increase to 2.5 million in the year 2035 [2]. The 5-year survival rate of CRC is around 90%, but it drops to around 10% for metastatic CRC [3]. The high mortality rates of CRC are due to the fact that 25% of CRC cases have metastases at the time of diagnosis and up to 50% of patients develop them after diagnosis [4].

Although new drugs against CRC have been developed in recent years, chemotherapy remains the mainstream of the first-line treatment [5]. Chemotherapy for CRC has undesirable side effects, which induce death of normal cells and reduce patients’ quality of life [6,7]. Hence, drugs targeting CRC effectively and without normal cell toxicity are urgently required to improve the patients’ quality of life. In previous studies, melatonin alleviated the undesirable side effects and enhanced the anticancer effects when used as an adjuvant therapy in existing anticancer treatment [8,9,10]. Studies proved that melatonin inhibits cell proliferation and induces apoptosis in breast cancer [11], induces anti-angiogenic effects in liver cancer [12], and inhibits invasion and migration in ovarian cancer [13]. Melatonin has shown promising anticancer effects by anti-inflammation and antioxidation in cancer cells [9,14]. Our previous publication reported that melatonin prevented oxidative stress-induced mitochondrial dysfunction in high glucose-treated Schwann cells, proving the antioxidant properties of melatonin [15]. Melatonin also increases DNA repair capacity in oxidant-challenged cells by preventing the initiation and progression of cancer directly as a free radical scavenger and indirectly as an antioxidant enzyme regulator [16]. Combining antioxidant vitamins A and E with irinotecan can increase its cytotoxic effect. However, when melatonin was combined with irinotecan, no significant increment of cytotoxic effect was reported [17], although melatonin is a well-documented antioxidant, and it is also a conditional prooxidant, depending on the dose, cell types, and drug–drug interactions. Several studies reported melatonin induces reactive oxygen species (ROS) accumulation in cancer cell, which leads to cancer cell death [18,19]. Hence, our study investigated the oxidation properties of melatonin in CRC cells.

Autophagy plays a critical role in cancer and its function in cancer is controversial [20]. Although autophagy is a stress-related cell adaptation mechanism to avoid cell death, occasionally, it may be an alternative death pathway called autophagic cell death [21]. In HCT116 colon cancer cells and DU145 prostate cancer cells, endoplasmic reticulum (ER)-stress-induced autophagy removes polyubiquitinated protein aggregates and reduces cellular vacuolization [22]. A study of sphingolipid homeostasis revealed that the ER-stress-induced autophagy together with the Akt pathway activation in a protein kinase R (PKR)-like endoplasmic reticulum kinase (PERK)-dependent manner counteracts the ER-stress-induced apoptotic signaling [23]. In contrast, bis(dehydroxy)curcumin induces apoptosis-independent autophagic cell death in cancer [24]. Hence, the interplay between apoptosis and autophagy is tissue- and condition-dependent. A deeper look into the role of autophagy in CRC treated with melatonin is required to further support our previous work and the use of melatonin in anticancer treatment [14].

It would be premature to use melatonin to treat CRC because the mechanisms underlying its anticarcinogenic properties need to be investigated before melatonin is used as a standalone anticancer drug or as an adjuvant to other anticancer drugs. In the present work, we investigated the underlying anticancer mechanism of melatonin using HT-29, SW48, and Caco-2 CRC cells; the mechanism included oxidative stress, ER stress, AMPK, and PI3K/Akt/mTOR pathways.

## 2. Results

### 2.1. The Effects of Melatonin and Tauroursodeoxycholic Acid (TUDCA) on Cell Viability of CRC Cells

**Figure 1:**
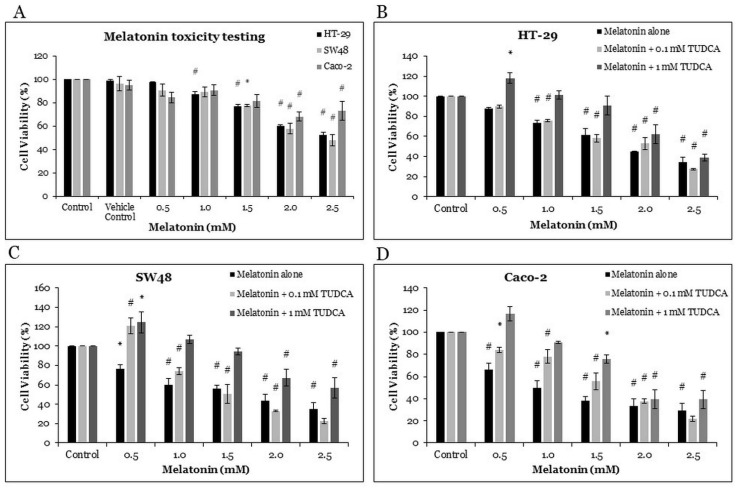
Cell viability assay: (**A**) HT-29, SW48, and Caco-2 were treated with various concentrations of melatonin for 72 h. (**B**–**D**) HT-29, SW48, and Caco-2 were treated with various concentrations of melatonin alone or together with 0.1 mM TUDCA or with 1 mM TUDCA. Means ± standard error of mean (SEM) of three separate experiments are shown. Two-way analysis of variance (ANOVA), followed by Dunnett’s post hoc test, was performed to compare each treatment group and control; statistically significantly different from control: *, *p* < 0.05; #, *p* < 0.01.

The HT-29, SW48, and Caco-2 cells were treated for 72 h with various concentrations of melatonin (0.5, 1.0, 1.5, 2.0, and 2.5 mM) and with vehicle control (98% ethanol, as (melatonin was dissolved in ethanol to a final concentration of 0.5%). The cell viabilities of HT-29, SW48, and Caco-2 were quantified with 3-(4,5-dimethylthiazol-2-yl)-2,5-diphenyltetrazolium bromide (MTT) assay (Figure 1A). The vehicle control and 0.5 mM melatonin had little inhibitory effect on the viability of the HT-29 cells. Melatonin alone significantly reduced the viability of the HT-29 cells in a dose-dependent manner (by 87, 77, 60, and 53% with 1.0, 1.5, 2.0, and 2.5 mM melatonin treatment, respectively). It also significantly reduced the viability of the SW48 cells (by 78, 58, and 48% with 1.5, 2.0, and 2.5 mM melatonin, respectively) and of the Caco-2 cells (68 and 73% with 2.0, and 2.5 mM melatonin, respectively).

In order to understand the role of ER stress in cell death, TUDCA, an ER stress inhibitor, was used in this study [25]. Trypan blue cell viability assay was performed for the HT-29, SW48, and Caco-2 cells treated with varying melatonin concentrations (0.5, 1.0, 1.5, 2.0, and 2.5 mM), as well as with their combinations with 0.1 mM TUDCA and 1 mM TUDCA. Melatonin alone induced significant cell death in all CRC cell lines, whereas TUDCA reversed melatonin-induced cytotoxic effects on all CRC cell lines; the potency of cytotoxic effects being melatonin alone > melatonin with 0.1 mM TUDCA > melatonin with 1 mM TUDCA (Figure 1C,D).

### 2.2. Melatonin Induces Oxidative Stress in HT-29 Cells

**Figure 2:**
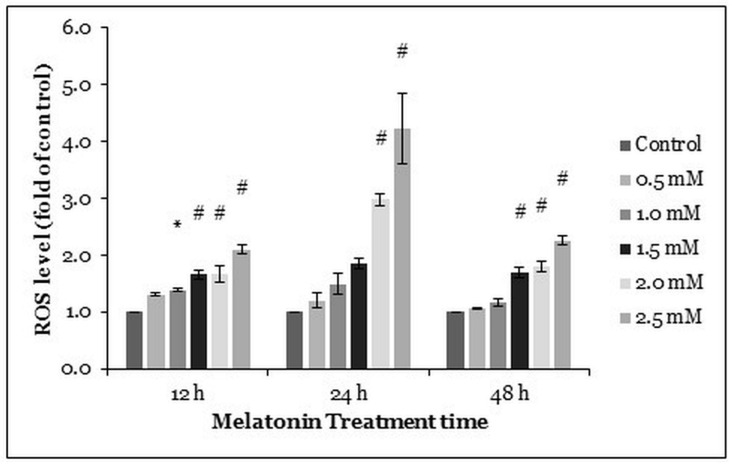
ROS generation in HT-29 cells after treated with various concentrations of melatonin for 72 h. The ROS generation was measured by DCFH-DA staining. Each bar represents mean ± SEM of three independent experiments. One-way ANOVA, followed by Dunnett’s post hoc test, was performed to compare each treatment group with the control. Statistically significantly different from the control: *, *p* < 0.05; #, *p* < 0.01.

We performed dichloro-dihydro-fluorescein diacetate (DCFH-DA) assay to quantify intracellular oxidative stress in the HT-29 cells after melatonin treatment (0.5, 1.0, 1.5, 2.0, and 2.5 mM) for 12, 24, and 48 h. Generally, the higher concentrations of melatonin increased ROS production in the HT-29 cells. At the 24 h time point, ROS levels in 2.0 and 2.5 mM melatonin-treated HT-29 cells were significantly elevated 2.99 and 4.22 times, respectively, as compared with the control. Thereafter, the ROS levels at 48 h post-treatment (significantly increased by ≥1.5 mM melatonin) were similar to the ROS levels at 12 h post-treatment (significantly increased by ≥1.0 mM melatonin) (Figure 2).

### 2.3. Melatonin Treatment Induces Autophagic Vacuoles Formation in HT-29 Cells

**Figure 3:**
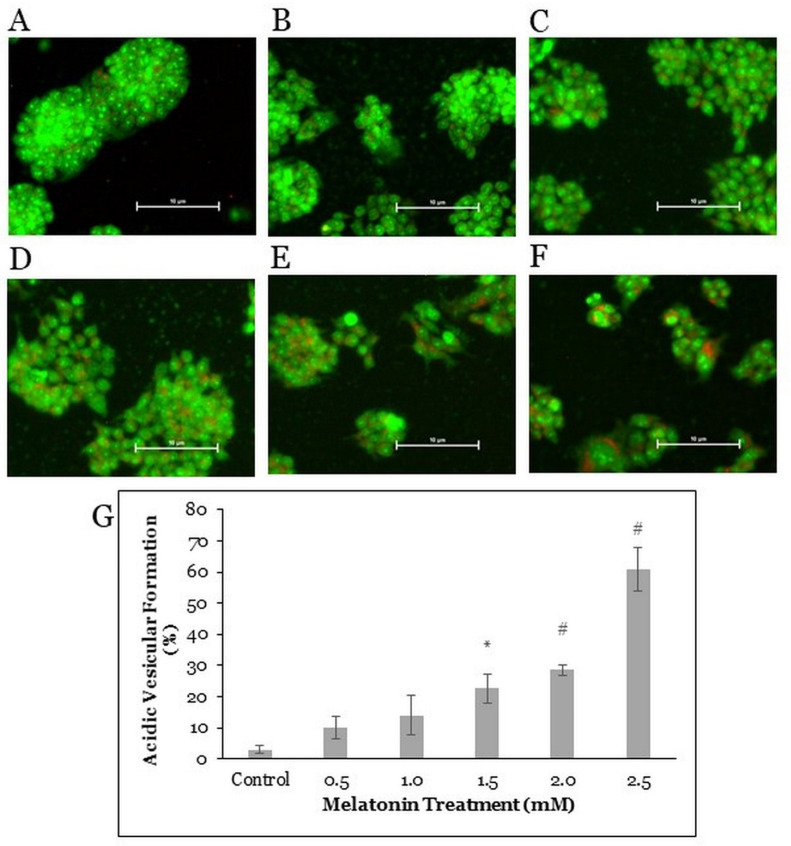
Acridine orange fluorescent staining in HT-29 cells. The HT-29 cells were treated with various concentrations of melatonin for 72 h: (**A**) control, (**B**) 0.5 mM, (**C**) 1.0 mM, (**D**) 1.5 mM, (**E**) 2.0 mM, and (**F**) 2.5 mM. Cells were stained with 5 μg/mL of the acridine orange dye for 20 min, then visualized under a fluorescence microscope with 200× magnification. (**G**) The bar graph represents the means ± SEM of three separate experiments. One-way ANOVA, followed by Dunnett’s post hoc test, was performed to compare each treatment group with the control. Statistically significantly different from control: *, *p* < 0.05; #, *p* < 0.01.

As autophagic cells form vacuoles, acridine orange forms aggregates that emit bright red fluorescence in acidic vesicles but emit green fluorescence in the nucleus and cytoplasm of the cells [26]. To further understand the mechanism of the HT-29 cell death induced by melatonin, fluorescent staining with acridine orange was done on the HT-29 cells after treatment with melatonin for 72 h (Figure 3A–F). The percentual ratios of bright red fluorescent cells to green fluorescent cells were evaluated. With the increasing concentration of melatonin, the percentage of autophagic vacuoles-positive cells increased, suggesting a dose-dependent mechanism of autophagy activation induced by the melatonin treatment. The proportions of autophagic vacuoles-positive cells after melatonin treatments were significantly different from those in control cells (22.7, 28.5, and 60.8% in case of 1.5, 2.0, and 2.5 mM melatonin, respectively) (Figure 3G).

### 2.4. Melatonin Alters ER Stress-Related Protein Expressions in HT-29 Cells

The PERK and serine/threonine-protein kinase/endoribonuclease (IRE1) activations depend on the binding immunoglobulin protein (BiP) activity. BiP is a sensor for unfolded proteins in the ER. When unfolded proteins accumulate during stress, BiP binds them, leaving PERK and IRE1 free to be activated. The activation of IRE1 could increase AMPK pathway activation and mammalian ortholog of the yeast autophagy-related gene 6 (Beclin-1) expression, leading to autophagy activation. The activation of PERK could induce autophagy-related proteins (ATGs) gene expression via regulation of C/EBP-homologous protein 10 (CHOP) transcription factors [27]. The eukaryotic translation initiation factor 2 subunit alpha (eIF2α) activation by PERK is also crucial for the ATGs protein expression and autophagosome formation [28]. Protein disulfide isomerase (PDI) is the PERK activator and the inactivated PDI reduces PERK signaling [29]. The expression level of calnexin increases in response to ER stress-causing stressors [30].

**Figure 4:**
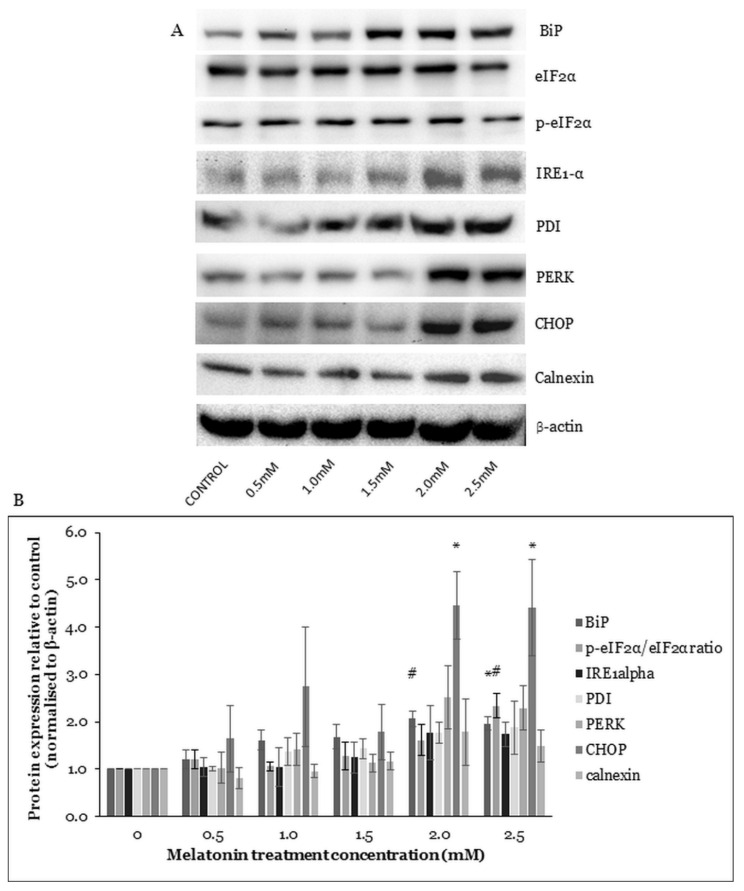
Western blot analysis of the ER stress in HT-29 cells after treated with various concentrations of melatonin for 72 h. (**A**) Expression levels of ER stress-related proteins were measured by Western blot analysis. Beta-actin (β-actin) served as a loading control. (**B**) Bar chart of the protein expressions; each bar represents a mean ± SEM of three independent experiments. One-way ANOVA, followed by Dunnett’s post hoc test, was performed to compare each treatment group with control; statistically significantly different from control: *, *p* < 0.05; #, *p* < 0.01.

To find out whether ER stress is affected in the HT-29 cells after treatment with melatonin, Western blotting was performed to study the protein expressions of ER stress-related proteins. Results suggested increased expressions of ER stress-related proteins BiP, IRE1-α, PDI, PERK, CHOP, and calnexin and an increased p-e-IF2α/e-IF2α ratio. Under the 2.0 and 2.5 mM melatonin treatments, the levels of BiP and CHOP were significantly increased, and under the 2.5 mM melatonin treatment, the p-e-IF2α/e-IF2α protein ratio was also statistically increased (Figure 4). The results indicated that melatonin induced the ER stress pathway activation in the HT-29 cells.

### 2.5. Melatonin Induces Changes in the AMPK Pathway

AMPK is a crucial cellular energy sensor protein and is activated by a low energy state in the cell. Phosphorylation of AMPK inhibits energy-consuming activities and promotes energy production under metabolic stress [31]. AMPK contributes to autophagosome maturation and lysosomal fusion [32]. Furthermore, the activation of AMPK downregulates the mTOR pathway to induce cell death [33].

**Figure 5:**
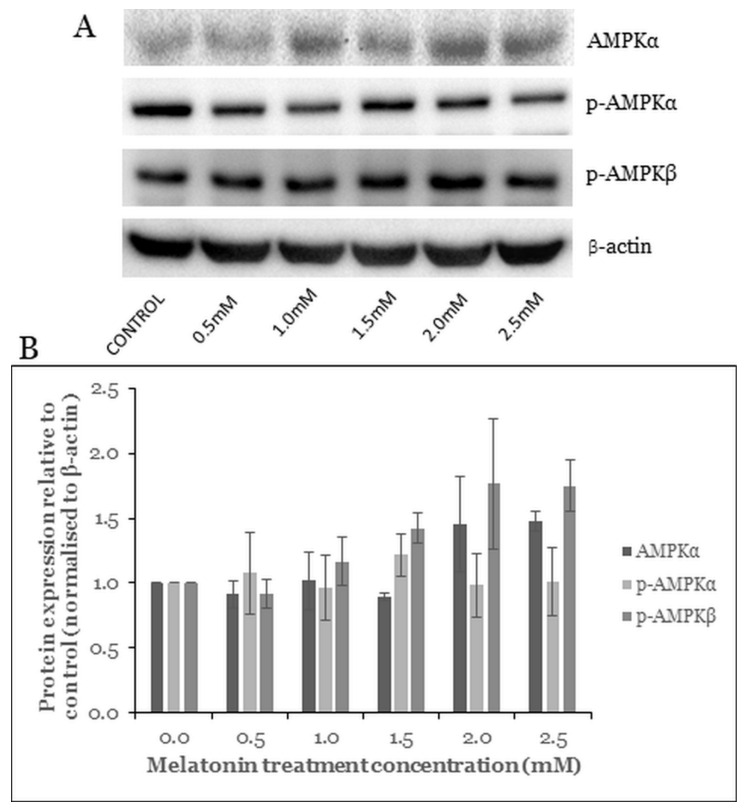
Western blot analysis of the AMPK pathway in HT-29 cells after treated with various concentrations of melatonin for 72 h. (**A**) Expression levels of AMPK proteins were assessed by western blot analysis. (**B**) Bar chart of the protein expressions; each bar represents a mean ± SEM of three independent experiments. One-way ANOVA, followed by Dunnett’s post hoc test, was performed to compare each treatment group with control.

To elucidate whether melatonin can affect the AMPK pathway in the HT-29 cells, Western blot analysis was carried out. Results showed an alteration in the level of the AMPK pathway proteins. The expression levels of the AMPKα, p-AMPKα, and p-AMPKβ increased with increasing concentration of melatonin. However, these changes were not statistically significant (Figure 5).

### 2.6. Melatonin Induces Changes in the PI3K/Akt/mTOR Pathway

**Figure 6:**
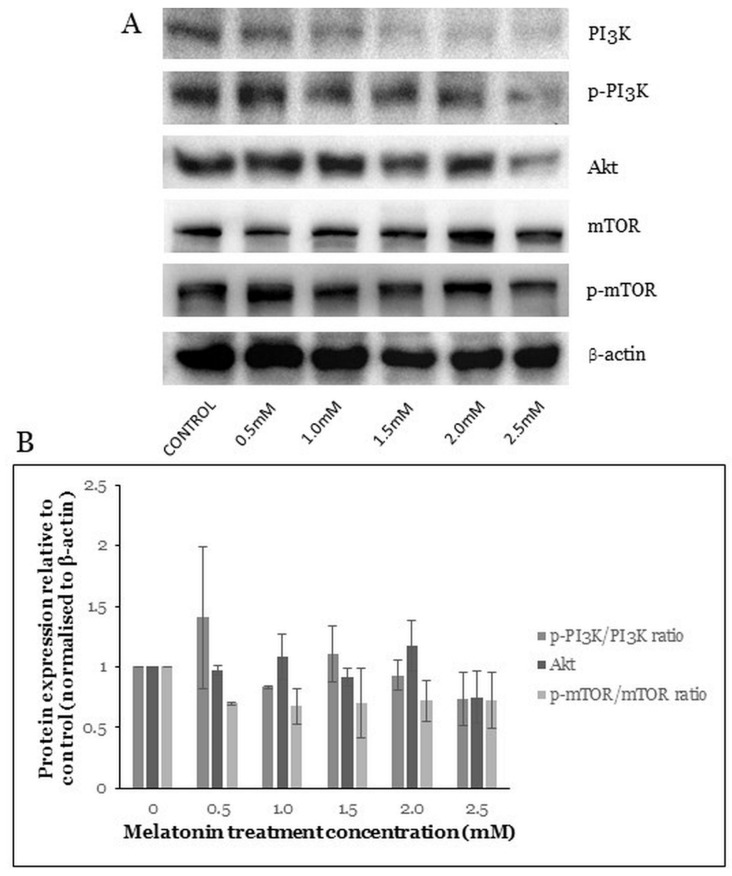
Western blot analysis of the PI3K/Akt/mTOR pathway in HT-29 cells after treated with various concentrations of melatonin for 72 h. (**A**) Expression levels of PI3K/Akt/mTOR proteins were assessed by Western blot analysis. (**B**) Bar chart of protein expressions; each bar represents a mean ± SEM of three independent experiments. One-way ANOVA, followed by Dunnett’s post hoc test, was performed to compare each treatment group with control.

The PI3K/Akt/mTOR pathway plays a critical role in controlling cell survival, proliferation, apoptosis, and autophagy [11]. Akt is directly activated by PI3K and is a major effector of PI3K involved in cancer growth. Akt signaling leads to increased cellular growth and survival. One of the major effectors downstream of Akt is mTOR [34]. In our study, the PI3K/Akt/mTOR pathway protein levels were studied after the HT-29 cells were treated with melatonin. Results showed changes in the levels of the PI3K/Akt, mTOR, and phosphorylated mammalian target of rapamycin (p-mTOR) proteins (Figure 6A). However, the changes in levels of the PI3K, phosphorylated phosphatidylinositol 3 kinase (p-PI3K), Akt, mTOR, and p-mTOR were not statistically significant (Figure 6B).

### 2.7. Melatonin Alters the Level of Autophagy Proteins

The formation of an autophagosome is a stepwise process, which involves initiation, nucleation, elongation, maturation, and degradation. The nucleation depends on Beclin-1 activation and its formation of complexes with other autophagy-related proteins. The following elongation involves Atg5 and the mammalian homologue of yeast ATG8 (LC3 protein family) [35]. Beclin-1, Atg5, and LC3 are commonly used autophagy markers for CRC study [36].

**Figure 7:**
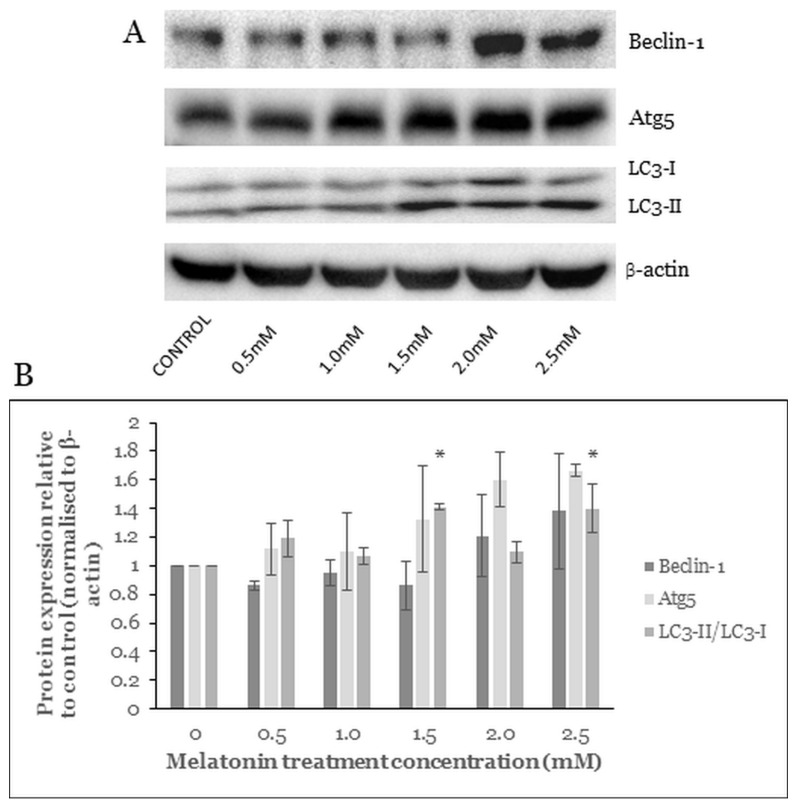
Western blot analysis of the melatonin-treated HT-29 cells. Effects of melatonin on autophagy pathway protein expressions in HT-29 cells after treated with various concentrations of melatonin for 72 h. (**A**) Expression levels of autophagy proteins were assessed by western blot analysis. (**B**) Bar chart of proteins expression; each bar represents a mean ± SEM of three independent experiments. One-way ANOVA was performed to compare each treatment group with control; statistically significantly different from control: *, *p* < 0.05.

Western blot analysis of the HT-29 cells treated with various melatonin concentrations showed that protein levels of the Beclin-1 and Atg5 were increased but not statistically significantly (Figure 7B). LC3-I (molecular weight of 16) is a cytoplasmic form processed into LC3-II (molecular weight of 14), which is autophagosome-membrane-bound. Hence the amount of LC3-II is correlated with the extent of autophagosome formation. In our study, after 72 h treatment of the HT-29 cells with melatonin, the LC3-II/LC3-I ratio was significantly increased at melatonin concentrations of 1.5 mM and 2.5 mM.

## 3. Discussion

The past decade has seen an enormous interest in melatonin due to its therapeutic potential in a variety of diseases, such as diabetes [37], intervertebral disc degeneration [38], cardiac anomalies [39], colitis [40], and cancer [41]. Melatonin induces both apoptosis and autophagy in Hodgkin lymphoma cells [42]. It also promotes synergistic cytotoxic effects with the chemotherapeutic drug sorafenib in hepatoma cancer cells [43]. Interestingly, autophagic vacuolization was reported to be necessary for the completion of apoptosis in another study [44]. Therefore, the study of cancer cell death should include both apoptosis and autophagy cell death mechanisms to gain a better understanding of pharmacodynamics of a particular drug. In the present study, we investigated the activation of autophagy in melatonin-treated HT-29, SW48, and Caco-2 cells, further supporting our previous work [14].

MTT cell viability assay was used to evaluate cytotoxic properties of melatonin. This method is a well-established colorimetric method for assessing cell viability and proliferation. After 72 h treatment, melatonin significantly inhibited the viability of the HT-29, SW48, and Caco-2 cells in a dose-dependent manner. It has been proven in our previous study that 72 h is the most effective time for killing cancer cells [14]. Moreover, Huang et al. (2020) also demonstrated that melatonin inhibited the survival of human gastric cancer cells under ER stress after 72 h of treatment [45] Using trypan blue cell viability assay, we found that the half-maximal inhibitory concentration (IC50) of melatonin for the HT-29, SW48, and Caco-2 cells were 1.89, 1.93, and 1.77 mM, respectively. Hence, treating these cells with melatonin concentration higher than 1 mM is necessary to achieve IC50, which is relevant for the potential of melatonin as a single anticancer agent. Moreover, only HT-29 showed a significant decrement of cell viability under 1.0 mM melatonin treatment, whereas SW48 and Caco-2 showed statistically significant decrement of cell viability at 1.5 mM and 2.0 mM, respectively. Our results are in agreement with those of Guangyu et al. (2021), who found that melatonin (0.1–2.0 mM) significantly inhibited CRC cell viability in a time- and dose-dependent manner [46]. Moreover, Farriol et al. proved that treatment with 3 mM melatonin could achieve the highest cell death (47%) in the CT-26 murine CRC cell line [47]. Generally, the trypan blue assay detected lower thyroid cancer-cell viability than the MTT assay, and the two assays were highly correlated (r = 0.99, *p* < 0.001) in the measurement of thyroid cancer-cell viability after melatonin treatment [48]. 

TUDCA is an effective ER stress inhibitor, which reduces ER stress-associated protein expression and ER stress-mediated cell death [49]. Hence, we selected TUDCA to assess the effects of melatonin-induced ER stress-mediated cell death. TUDCA attenuated the melatonin-mediated cell death in all CRC cell lines and TUDCA attenuated it stronger at 1 mM than at 0.1 mM. The cytotoxic effects of melatonin were in this descending order: melatonin alone > melatonin with 0.1 mM TUDCA > melatonin with 1 mM TUDCA (Figure 1B–D). Thus, we conclude that melatonin-mediated CRC cell death is dependent on ER stress.

Autophagy is closely related to oxidative stress in which redox signaling regulates the autophagy activities and autophagy regulates oxidative stress levels with the participation of mitochondria and the activation of mitophagy [50]. Research found that accumulation of ROS, membrane lipid oxidation, and loss of plasma membrane integrity are the main causes of autophagy. Catalase, the leading ROS scavenger, is selectively degraded in the autophagy process, leading to abnormal ROS accumulation. Caspases directly cause catalase degradation and ROS accumulation, which can be prevented by autophagy inhibitors [51]. Melatonin was reported to induce cancer cell death through a calmodulin-dependent ROS production [18]. We speculate that melatonin might induce CRC cell death via a mechanism similar to that of prooxidants. Apart from that, several anticancer therapeutic agents have been developed that promote oxidation in cancer cells. For instance, curcumin induced ROS accumulation, ER stress upregulation, and vacuolated cell death, which was also reported in our previous research about the HT-29 cells treated with melatonin [52]. Delicaflavone, a novel anticancer agent, a biflavonoid from *Selaginella doederleinii Hieron*, induced ROS accumulation and inhibited the PI3K/Akt/mTOR signaling pathway, demonstrating the importance of studying ROS and the PI3K/Akt/mTOR pathway in the context of cancer [53].

The formation of autophagic vacuoles is a well-established feature of autophagic cells [26,54]. Acridine orange forms aggregates that emit bright red fluorescence in acidic vesicular organelles and green fluorescence in the cytoplasm and nucleus [26]. Acridine orange staining is a quick, accessible, and reliable method to determine the amount of acidic vesicular organelles, which increases upon autophagy induction, and ratiometric analysis of acridine orange staining is a well-established method in the study of autophagy. Lysosomal degradation of unwanted organelles is the last step in the autophagy, an indication of autophagy activation and prerequisite for autophagic cell death [45,55,56]. Based on our data, melatonin induces autophagy and autophagic vacuoles formation in the HT-29 cells in a dose-dependent manner. The percentage of autophagic vacuoles-positive cells was significantly different from control (22.7, 28.5, and 60.8% after 1.5, 2.0, and 2.5 mM melatonin treatment, respectively).

A variety of physiological and pathological conditions can lead to the accumulation of misfolded proteins in the ER, leading to ER stress. The unfolded protein response (UPR) attenuates the ER stress and re-establishes protein homeostasis. The UPR-signaling pathway is overexpressed in various types of tumors and plays a key role in tumor growth, adaptation, and resistance to cancer treatment. However, UPR activation promotes cell death during prolonged ER stress [28]. A previous study reported that melatonin activated autophagy by controlling ER stress in gastric cancer cells [57]. Similarly as in our study, the ER stress-related genes expressions were increased after melatonin treatment, indicating that autophagy was induced by ER stress. Calnexin is a calcium-binding protein that keeps newly synthesized glycoproteins inside the ER to ensure proper protein folding and to ensure protein quality [58]. In our study, BiP and CHOP protein levels were significantly increased. Increased phosphorylation of eukaryotic translation initiation factor 2 subunit alpha (p-eIF2α) was reported after melatonin treatment, which may have been related to increased ER stress. ER associates with early autophagic structures called isolation membranes (IMs) [28]. The promoted release of BiP and activation of the eIF2α/CHOP pathway could induce autophagy [59], in agreement with our Western blot results that suggested upregulation of BiP, p-eIF2α/eIF2α, and CHOP expressions. The PERK/CHOP pathway could induce ATGs protein expressions followed by autophagosome formation [27]. We observed an increase in PERK (not statistically significant, *p* = 0.066 and 0.133 at 2.0 and 2.5 mM melatonin, respectively) and CHOP (statistically significant, *p* = 0.040 and 0.043 at 2.0 and 2.5 mM melatonin, respectively); therefore, the PERK/CHOP pathway could potentially contribute to melatonin-mediated CRC cell death via autophagy.

AMP-activated protein kinase (AMPK) is an important energy sensor and is activated by low energy availability in the cell [60]. In addition, AMPK activates tuberous sclerosis complex 2 (TSC2), which in turn suppresses mTOR complex 1 (mTORC1), thus promoting autophagosome formation [61]. A study reported that the AMPK contributes to autophagosome maturation and autophagosome–lysosome fusion [32]. Combined treatment using melatonin and 5-fluorouracil in CRC stem cells increased the phosphorylation of AMPK but decreased the phosphorylation of mTOR [62]. However, we observed only a statistically insignificant increase in AMPKα and p-AMPKβ, whereas p-AMPKα remained unchanged for all melatonin treatment concentrations.

The PI3K/Akt pathway plays a major role in regulating cell survival, proliferation, and apoptotic cell death. Melatonin was shown to inhibit breast cancer cell proliferation via the PI3K/Akt pathway [11]. The co-treatment of melatonin with ER stress inducers promoted melanoma cell death by suppressing the PI3K/Akt pathway [63]. The inactivated mTOR promotes autophagy, and the mTOR also serves as a central cell-growth regulator that integrates growth factors and nutrient signals. The AMPK and the PI3K/Akt pathways converge on mTOR with opposing regulatory effects; AMPK regulates mTOR negatively while PI3K/Akt positively [64]. In our study, we observed a decrease in the p-PI3K/p-PI3K ratio, Akt, and p-mTOR/mTOR protein ratio but not statistically significant.

The autophagy pathway requires five steps: initiation, nucleation, elongation, maturation, and degradation with the involvement of numerous Atg and non-Atg proteins. Beclin-1, Atg5, and LC3-II are the commonly used indicators for autophagy [36]. Based on our study, the upregulation of Beclin-1, Atg5, and LC3-II/LC3-I ratio were observed in HT-29 cells after melatonin treatment for 72 h. Beclin-1 involves every major step in autophagic pathways, from autophagosome formation to the maturation of the autophagosome/endosome [35]. In a previous study, ER stress inducers activated the Beclin-1, Atg5, and LC3-II proteins and induced cell death in colon and prostate cancer. The ER stress inducers caused cancer cell death in an Atg5-dependent manner, in accordance with our results [22]. A betulinic acid (BA) analogue, 2c, caused autophagic cell death in CRC cells via upregulation of Beclin-1, Atg5, and LC3 [26]. A similar autophagic cell death mechanism was also observed in A375 and CHL-1 cell lines, ophiobolin A increases ROS accumulation and LC3-II protein expression to induce autophagic cell death in human melanoma cells [65].

Autophagy is a complicated and dynamic intracellular mechanism, which has multiple variations, such as chaperone-mediated autophagy, microautophagy, and macroautophagy. Our study focused on the macroautophagy, investigation of the other variations of autophagy could potentially provide an extensive understanding on how melatonin induces autophagic cell death in CRC. Moreover, there are also canonical and non-canonical autophagy pathways that require further investigation [64]. A comprehensive mapping of the autophagy network could provide us with an anticancer target and even previously unknown molecular targets for various diseases.

## 4. Materials and Methods

### 4.1. Materials

Dulbecco’s modified Eagle medium (DMEM), fetal bovine serum (FBS), MTT, melatonin, and acridine orange were purchased from Sigma Aldrich (Saint Louis, MO, USA). TUDCA was purchased from Merck Millipore (Burlington, MA, USA). Trypsin–ethylenediaminetetraacetic acid (EDTA) was purchased from Gibco (Gibco, Loughborough, UK). Dimethyl sulfoxide (DMSO) and bovine serum albumin (BSA) were purchased from Nacalai Tesque (Kyto, Japan). Primary antibodies against β-actin, PI3K, p-PI3K, Akt, AMPKα23a, p-AMPKα, AMPKβ, autophagy-related protein Atg5, Beclin-1, BiP, calnexin, eIF2α, p-eIF2α, IRE1α, PDI, microtubule-associated protein 1 light chain 3 (LC-3), mTOR, p-mTOR, PERK, and CHOP and horseradish peroxidase-conjugated secondary antibodies were purchased from Cell Signalling Technology (Danvers, MA, USA). SuperSignal^TM^ West Femto Maximum Sensitivity Substrate was purchased from Thermo Fisher Scientific (Waltham, MA, USA). Melatonin was dissolved in 98% absolute ethanol; the stock concentration of melatonin was maintained at 500 mM for every experiment. The final volume of ethanol in cell culture was maintained below 0.5%. TUDCA was dissolved in phosphate-buffered saline (PBS), the stock concentration of TUDCA was maintained at 100 mM for every experiment.

### 4.2. Cell Culture

HT-29, SW48, and Caco-2 were obtained from the American Type Culture Collection (ATCC). The cells were cultured in DMEM and supplemented with 10% FBS at 37 °C in an incubator with 5% CO_2_. Cells were trypsinized whenever the confluency of cells reach 70%. Cells collection was carried out by rinsing the cells with PBS Biobasic (Toronto, ON, Canada) followed by the addition of trypsin-EDTA to detach the cells. The action of trypsin was later neutralized with DMEM, and cells were harvested by centrifugation at 1500 rpm for 5 min. The cells were then sub-cultured into new cell culture T-25 flasks or plated for assays.

### 4.3. MTT Cytotoxicity Assay

The cytotoxicity of melatonin was measured in the HT-29, SW48, and Caco-2 cells by the MTT colorimetric assay. The CRC cells were seeded at 5000 cells (in 100 μL culture medium) per well in 96-well plates and incubated for 24 h. Subsequently, the cells were treated with melatonin at various concentrations (0, 0.5, 1.0, 1.5, 2.0, and 2.5 mM). After an incubation for 72 h, the cells were incubated with 20 µL of MTT (5 mg/mL) solution for 4 h. Following medium removal, 100 µL of DMSO was added to each well and plates were gently shaken. The optical absorbance was measured at 570 nm, with 630 nm as reference wavelength, using Spectra Max3 Molecular Devices (San Jose, CA, USA). The absorbance of cells without treatment was regarded as 100% cell survival. Each experiment was repeated at least three times, beginning from the cell seeding to data analysis.

### 4.4. Trypan Blue Cell Viability Assay

The cytotoxicity of melatonin was measured in the HT-29, SW48, and Caco-2 cells by trypan blue dye exclusion assay. The CRC cells were seeded at 3.0 × 10^4^ cells (in 5000 μL culture medium) per well in 60 mm^2^ dishes and incubated for 24 h. Then, the cells were treated with various melatonin concentrations (0, 0.5, 1.0, 1.5, 2.0, and 2.5 mM), various melatonin concentrations + 0.1 mM TUDCA, and various melatonin concentrations + 1 mM TUDCA. After incubation for 72 h, the cells were trypsinized and collected in 15 mL tubes. The cells were stained with trypan blue for cell count using a hemocytometer and light microscope. Each experiment was repeated at least three times, beginning from the cell seeding to data analysis.

### 4.5. ROS Level Analysis

The HT-29 cells were cultured in 96-well plates, then treated with or without melatonin (0.5, 1.0, 1.5, 2.0, and 2.5 mM) for 12, 24, and 48 h. The cells were stained with 10 µM of DCFH-DA for 30 min. The cells were then examined under a microplate reader Spectra Max3 Molecular Devices (San Jose, CA, USA). Fluorescence intensity in the cells was detected at an excitation wavelength of 485 nm and an emission wavelength of 535 nm. Each experiment was repeated at least three times, beginning from the cell seeding to data analysis.

### 4.6. Fluorescence Staining

Lysosomal activation was detected by acridine orange fluorescent staining and microscopy. The HT-29 cells (1.0 × 10^5^) were seeded onto a 60 mm^2^ dish for 24 h (37 °C, 5% CO_2_). The cells were then treated with melatonin at various concentrations (0, 0.5, 1.0, 1.5, 2.0, and 2.5 mM). After 72 h, the cells were washed with 200 µL of PBS and stained with Acridine orange (5 µg/mL) for 15–20 min. The cells were observed under an inverted fluorescence microscope Nikon Eclipse Ti (Tokyo, Japan). The activation of lysosomes was examined, and photomicrographs were taken using an attached camera. Each experiment was repeated at least three times, beginning from the cell seeding to data analysis.

### 4.7. Western Blot Analysis

Lysates of the cells exposed to melatonin for 72 h were harvested using DTT lysis buffer (62.5 mM Tris, 2% *w/v* SDS, 10% glycerol, pH 6.8, 100 mM DTT). The protein concentration was determined using Quick Start™ Bradford Protein Assay (Bio-Rad, Hercules, CA, USA). From each sample, 15 µL of proteins were separated by electrophoresis, on 7.5, 10, and 12.5% SDS-PAGE gels, based on the molecular weight of proteins. After the electro-transfer of proteins onto PVDF membranes, the membranes were blocked with 3% BSA for 1 h at room temperature. The membrane was then incubated with primary antibodies at a dilution of 1:1000, overnight at 4 °C. After three washes with TBST (0.1% Tween-20 in Tris-HCl buffered saline, TBS), the membranes were incubated with horseradish peroxidase-conjugated secondary antibodies at a dilution of 1:10,000, for 1 h at room temperature. After washing with TBST and TBS, proteins were visualized by SuperSignal^TM^ West Femto Maximum Sensitivity Substrate. Densitometric analysis was performed using ImageLab, Version 6.1.0, build 7 Standard Edition (Bio-Rad, Hercules, CA, USA) to scan the signals. Western blot assay results reported here are representative of at least three independent experiments (repeated from the cell seeding to data analysis).

### 4.8. Statistical Analysis

All experiments were performed in biological triplicates and values given are representative for at least three independent experiments. Each result of the experiments was expressed as mean ± standard error of mean (SEM). The results were statistically analyzed by one-way or two-way ANOVA followed by Dunnett’s post hoc test using IBM SPSS Statistics, Version 23. Values *p* < 0.05 or *p* < 0.01 were considered as statistically significant.

## 5. Conclusions

In conclusion, our results indicate that melatonin induces an anticancer effect in the HT-29, SW48, and Caco-2 CRC cells via oxidative stress-mediated autophagy. Melatonin-induced autophagy involves the ER stress signaling pathway. With the effects of these pathways, stepwise autophagosome formation was confirmed via Beclin-1 (nucleation), Atg5 (elongation), and LC3 (elongation). Our study provided an insight into the underlying mechanism of melatonin-induced autophagy and its pro-oxidative properties in CRC cell.

## Data Availability

Not applicable.

## Sample Availability

Samples of the compounds are not available from the authors.
